# Serological biomarkers in autoimmune GFAP astrocytopathy

**DOI:** 10.3389/fimmu.2022.957361

**Published:** 2022-08-02

**Authors:** Cong-Cong Fu, Lu Huang, Lu-Fen Xu, Li-Hong Jiang, Hui-Lu Li, Sha Liao, Jiajia Yue, Chun Lian, Xin-Guang Yang, You-Ming Long

**Affiliations:** ^1^ Department of Neurology, The Second Affiliated Hospital of Guangzhou Medical University, Guangzhou, China; ^2^ Department of Neurology, Institute of Neuroscience, Key Laboratory of Neurogenetics and Channelopathies of Guangdong Province and the Ministry of Education of China, The Second Affiliated Hospital, Guangzhou Medical University, Guangzhou, China; ^3^ Department of Neurology, Sun Yat-Sen Memorial Hospital, Sun Yat-Sen University, Guangzhou, China

**Keywords:** GFAP-A, autoantibody, autoimmune encephalitis, MIP 3 alpha, CSF abnormalities

## Abstract

Autoimmune glial fibrillary acidic protein astrocytopathy (GFAP-A) is a newly defined meningoencephalomyelitis. The pathogenesis of GFAP-A is not well understood. The present study measured the expression levels of 200 serological cytokines in GFAP-A patients, NMOSD patients and healthy controls (HCs). The correlations between serum cytokine levels and clinical information in GFAP-A patients were analyzed. A total of 147 serological proteins were differentially expressed in GFAP-A patients compared to HCs, and 33 of these proteins were not observed in NMOSD patients. Serum levels of EG-VEGF negatively correlated with GFAP antibody titers, MIP-3 alpha positively correlated with clinical severity in GFAP-A patients, and LIGHT positively correlated with WBC counts and protein levels in the CSF of GFAP-A patients. These results suggest that GFAP and AQP4 astrocytopathy share some common pathology related to TNF signaling. Serum MIP 3 alpha may be a biomarker to assess clinical severity and a potential target for therapy of autoimmune GFAP astrocytopathy.

## Introduction

Autoimmune glial fibrillary acidic protein astrocytopathy (GFAP-A) is an autoimmune inflammatory central nervous system (CNS) disorders that was defined by Fang et al. in 2016 (1). Identification of cerebrospinal fluid (CSF) GFAP-IgG using tissue-based assay (TBA) and cell-based assay (CBA) helps the diagnosis of GFAP-A. GFAP-A generally involves the cerebra, meninges, spinal cord and brain, and clinical manifestations include fever, headache, encephalopathy, myelitis, and abnormal vision. The characteristic MRI feature of GFAP-A is brain linear perivascular radial gadolinium enhancement in the white matter perpendicular to the ventricle. Although CSF GFAP antibody is a good biomarker for the diagnosis of GFAP-A, it cannot induce pathological changes. The pathogenesis of GFAP-A is not well understood, and the pathology of GFAP-A in humans is heterogeneous. Previous studies suggested that the presence of CD8+ T cells was an important pathological diagnostic feature of GFAP-A (2, 3). Other studies speculated that other inflammatory components of the immune system contributed to GFAP-A pathogenesis (4, 5). The presents study measured the 200 serum protein concentrations of healthy controls (HCs), GFAP-A patients and neuromyelitis optica spectrum disorder (NMOSD) patients using antibody arrays. Differential expression proteins (DEPs) were analyzed between GFAP-A and HCs, NMOSD and HCs, and GFAP-A and NMOSD. We examined the correlations between serological protein levels, CSF anti-GFAP antibody titers and clinical severity. Altogether, our data provide further insight into the pathogenesis of GFAP-A and identify potential biomarkers that may aid in the more accurate diagnosis of GFAP-A patients.

## Materials and methods

### Study participants

The committees for ethical review of research involving human subjects at the Second Affiliated Hospital of Guangzhou Medical University (Guangzhou, China) approved this study. All patients provided written informed consent. A total of 28 healthy controls (HCs), 28 NMOSD patients who fulfilled the diagnostic criteria (6), and 14 GFAP-A patients (1, 7) were enrolled in the study. All serum samples were processed following standard procedures and frozen at -80°C until use. CSF samples from 14 GFAP-A patients were collected and routinely tested for autoantibodies. Serum autoantibodies against AQP4, MOG and GFAP-A were detected using cell-based assays as previously described (8–10). All NMOSD patients were seropositive for AQP4 autoantibodies, and NMOSD and GFAP-A patients were seronegative for anti-MOG autoantibodies.

### Detection of cytokine concentrations

The concentrations of 200 serum proteins were measured simultaneously using the Human Cytokine Array Q4000 (QAH-CAA-4000, RayBiotech, Georgia, USA) as previously described (11). Briefly, after incubated with blocking buffer for 60 min at room temperature (RT), the arrays were added with 100 µL of 2-fold diluted serum and were incubated at 4°C overnight. On day 2, the arrays were added biotin-labeled detection antibody for 1 hour (h) at RT followed by extensive washing. Alexa Fluor 555-conjugated streptavidin was added and incubated for 1 h at RT. The signals were scanned, and data were extracted by an InnoScan 300 scanner (Innopsys, Carbonne, France).

### Protein-Protein-Interaction (PPI) network construction

The PPI network was constructed in STRING, which is a useful online database for constructing PPI networks. To evaluate their interactive associations, all DEPs were entered into the STRING database, and the interaction network, Kyoto Encyclopedia of Genes and Genomes (KEGG) pathway analysis and biological process (BP) analysis were further analyzed in STRING.

### Data processing, figure generation and statistical analysis

The methods of data acquisition/processing, figure generation and statistical analysis of protein arrays was referred to the previous study (11). The relationship between CSF levels of WBC, protein, chloride, glucose and serum concentrations of 200 proteins were calculated using Pearson’s correlation coefficients in SPSS 20.0 software (SPSS, Inc., Chicago, USA). The relationship between serum concentrations of 200 proteins and GFAP antibody titers and the modified Rankin Scale (mRS) were calculated using Spearman’s correlation coefficients in SPSS 20.0 software. A p value <0.05 was considered statistically significant.

## Results

### Patient demographics and clinical data

A total of 28 HCs, 14 GFAP-A patients and 28 NMOSD patients who fulfilled the diagnostic criteria were enrolled in the study. Patient demographic and clinical characteristics are described in **Table 1**. The median age of HCs was 44 years (range, 22-63 years), GFAP-A patients was 48.5 years (range, 27-69 years) and NMOSD patients was 36 years (range, 22-72 years). For the disease duration, 9 NMOSD patients (32.1%) and one GFAP-A patient (7.2%) were longer than 12 months, and 19 NMOSD patients (67.8%) and 13 GFAP-A patients (92.8%) were shorter than 12 months. For the MRI features, 13 NMOSD patients (46.4%) and 10 GFAP-A patients (71.4%) showed brain lesions, and 25 NMOSD patients (89.2%) and 4 GFAP-A patients (28.5%) showed spinal cord lesions.

**Table 1:** Clinical information presentation of HCs, NMOSD patients, and GFAP patients.

	HCs	NMOSD	GFAP
**Age (**Median ± SEM**)**	44.6 ± 12.8	38.2 ± 14.6	46.6 ± 11.3
**Number**	28	28	14
**Sex, female:male**	18:10	27:1	5:9
**Disease duration, n (%)**			
<12 months	/	19 (67.8)	13 (92.8)
≥12 months	/	9 (32.1)	1 (7.2)
**MRI features**			
Brain lesions, n (%)	/	13 (46.4)	10 (71.4)
Spinal cord lesions, n (%)	/	25 (89.2)	4 (28.5)

The GFAP-IgG titers and detailed information of 14 GFAP-A patients were showed in Supplementary **Table 1**. Representative images of CSF GFAP antibody detection by CBA were showed in **Supplementary Figure 1A**. This cohort was comprised of 5 females and 9 males. CSF GFAP antibodies were detected using cell-based assays as previously described (7), and GFAP-IgG titers ranged from 1:10 to over 1:1000. Among these 14 participants, one patient was positive for AQP4 antibody, one patient was positive for Scl-70 antibody, and two patients were positive for NMDAR and ITRP1. The main brain symptoms observed included fever (n= 7), headache (n = 8), altered consciousness (n = 4), psychosis (n = 4), weakness of limbs (n=4), jerk (n=1), vertigo (n = 3), dysuria (n=2), blurred vision (n=2), diplopia (n=2), ataxia (n=1), tremors (n=1), bradykinesia (n=1), fatigue (n=1), and myelitis (n=1). Eleven of the 14 participants received brain MRI scans. 10 of these patients showed brain abnormalities (90.9%), 4 patients showed spinal cord abnormalities (36.3%), and 1 patient showed meningeal abnormality (9.1%). Brain MRI in four patients (36.4%) revealed characteristic radial enhancing patterns. 4 of these patients (36.4%) exhibited lesions in the spinal cord, and 1 patient had very long lesions from the cervical segment to the lumbar segment of the spinal cord. CSF abnormalities were found in all 14 GFAP-A patients.

### Protein profiling using antibody arrays

**Figure 1:**
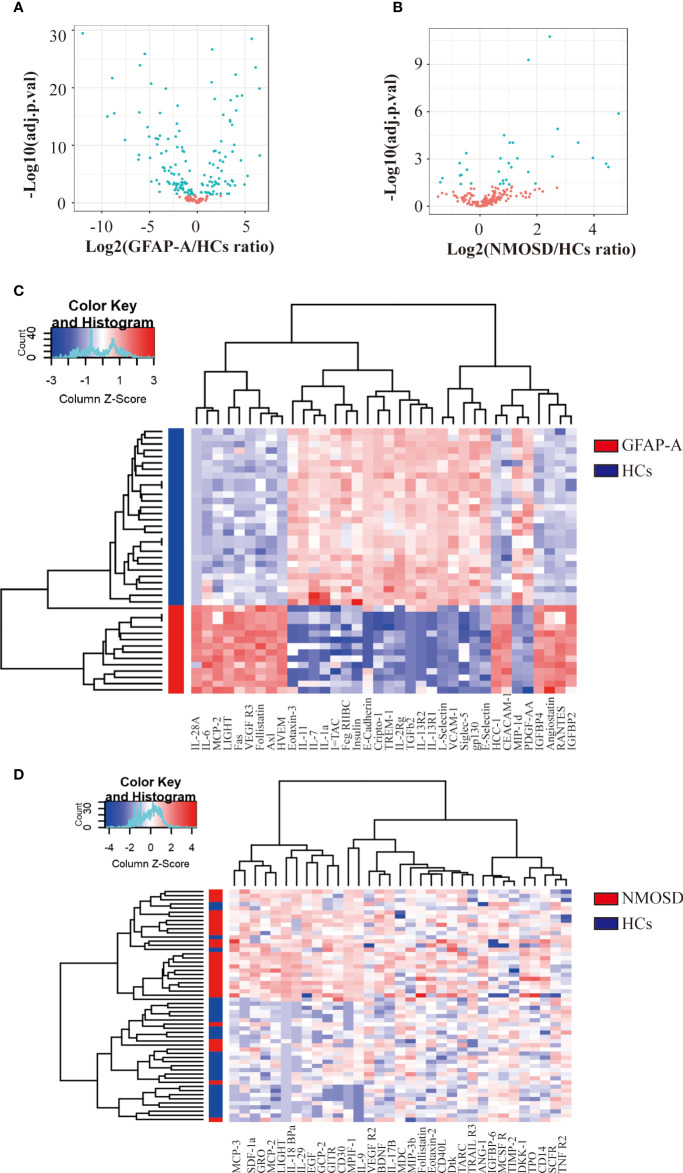
Antibody array analysis shows DEPs between HCs and NMOSD patients. **(A)** Volcano plot presented the distribution of 200 serum protein expression levels between GFAP-A patients and HCs. **(B)** Volcano plot presented the distribution of 200 serum protein expression levels between NMOSD patients and HCs (adjusted *p* < 0.05 and absolute log2 FC > 0.263). adj.P.val = adjusted p value. **(C)** Clustering heatmap of the top 30 DEPs between GFAP-A patients (red) and HCs (blue). **(D)** Clustering heatmap of the 33 DEPs between NMOSD patients (red) and HCs (blue).

To determine the protein biomarkers that were associated with GFAP-A, we used antibody arrays to detect the quantitative levels of 200 serological proteins of 28 HCs, 14 GFAP-A patients, and 28 NMOSD patients. A total of 147 cytokines were differentially expressed between HCs and GFAP-A patients (**Figure 1A**, volcano plot). The top 30 DEPs were clustered and are shown in the correlation heatmap (**Figure 1C**). A total of 33 cytokines were differentially expressed between HCs and NMOSD patients (**Figure 1B**, volcano plotting). All of the differentially expressed proteins were clustered and are shown in the correlation heatmap (**Figure 1D**).

**Figure 2:**
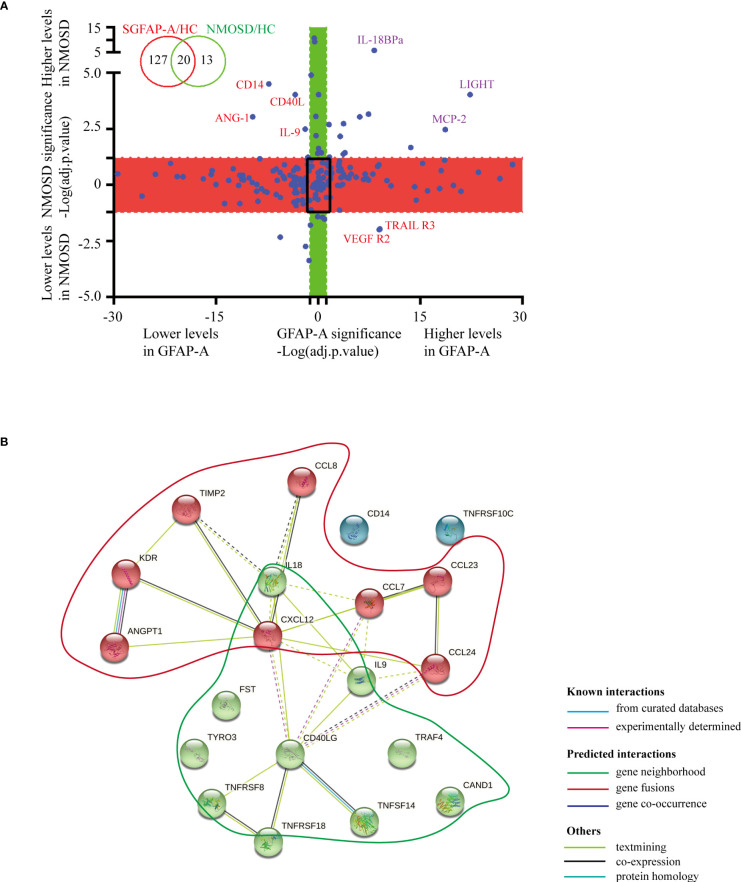
Serological protein profiles of GFAP and NMOSD patients reveal disease-common and disease-specific information. **(A)** Plot of the log10-transformed adjusted p value (adj.P.val) of GFAP-A and NMOSD data show preserved directionality, which resulted in the classification of four distinct groups: “Both changed” (adj.P.val _GFAP-A_ < 0.05 and adj.P.val _NMOSD_ < 0.05, same direction of changes in both diseases); “Changed in GFAP-A compared to NMOSD” (adj.P.val _GFAP-A_ < 0.05, adj.P.val _NMOSD_>0.05, red box); “Changed in NMOSD compared to GFAP-A” (adj.P.val _NMOSD_ < 0.05, adj.P.val _GFAP-A_ > 0.05, green box); “Non-significant” (adj.P.val _GFAP-A_ and adj.P.val _NMOSD_ > 0.05, black frame). **(B)** STRING analysis of 20 DEPs in the GFAP-A and NMOSD patient groups compared to HCs. A total of three clusters were identified with known/predicted/other interactions. Cluster 1, represented with 9 red balls, and was centered in CD40LG. Cluster 2, represented with 8 green balls, was centered on CXCL12.

When we plotted the log10-transformed adjusted *p*-values of GFAP-A and NMOSD patient data, there were 20 DEPs in the GFAP-A and NMOSD patient groups compared to HCs, including LIGHT, MCP-2, follistatin, ANG-1, TARIL R3, VEGF R2, IL-18 Bpa, GITR, CD14, Dtk, SCF R, CD30, eotaxin-2, MCP-3, CD40 L, SDF-1a, IL-9, TNF RII, MPIF-1 and TIMP-2 (**Figure 2A**, purple font and red font). Most of the DEPs had a same trend for the NMOSD and GFAP-A patient groups, respectively, except CD14, ANG-1, CD40 L, IL-9, VEGF R2 and TRAIL R3, which had an opposite trend between NMOSD patients and GFAP-A patients (**Figure 2A**, red font).

The interactions of the 20 DEPs in the GFAP-A and NMOSD patient groups (both DEPs) were explored using the STRING online database to investigate the PPI network that was likely associated with autoimmune astrocytopathy. As shown in **Figure 2B**, a total of three clusters were identified with known/predicted/other interactions. Cluster 1, represented with 9 red balls, was centered on CD40LG and was primarily enriched in the tumor necrosis factor-mediated signaling pathway and regulation of T-cell activation. Cluster 2, represented with 8 green balls, was centered on CXCL12 and was primarily enriched in eosinophil/lymphocyte/monocyte chemotaxis (Additional BP and KEGG information is shown in **Supplementary Table 2**). These results indicated that NMOSD astrocytopathy and GFAP astrocytopathy may share some common serological cytokine mechanisms.

**Figure 3:**
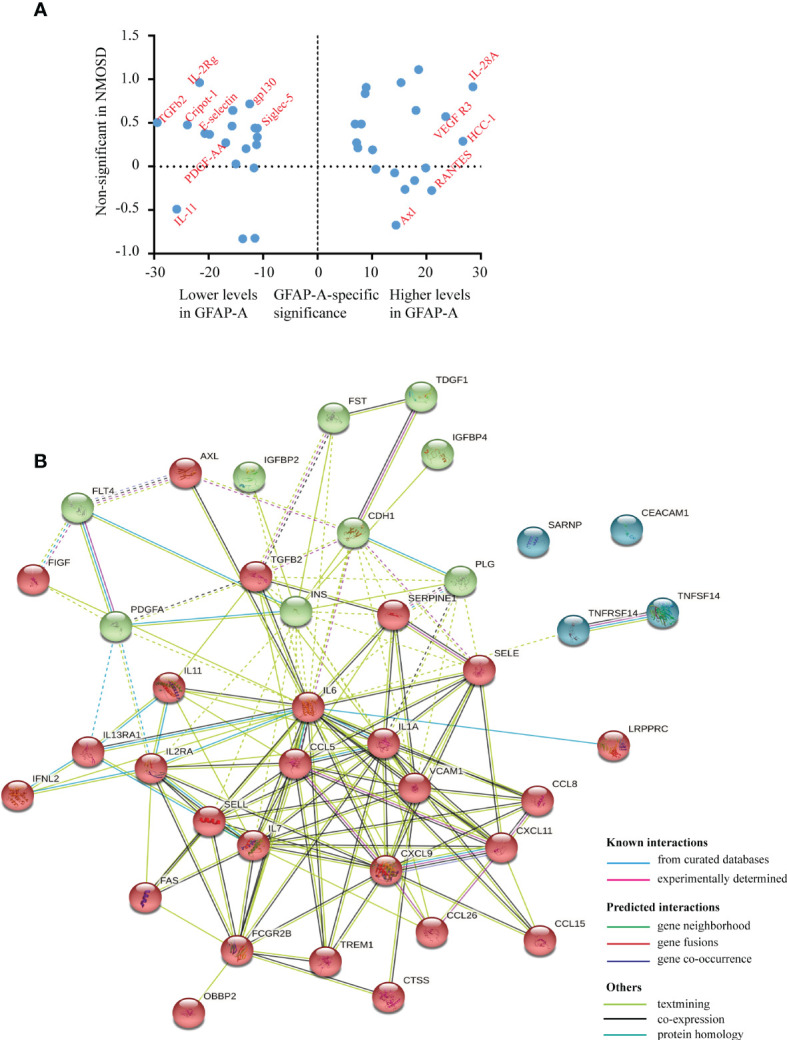
Serological protein profiles of GFAP-A patient disease-specific information. **(A)** Plot of the log10-transformed adjusted p value (adj.P.val) of the top 20 upregulated DEPs and top 20 downregulated DEPs unique to GFAP-A patients. The upregulated DEPS, such as VEGF R3, HCC-1, RANTES and Axl, are labeled on the right. The downregulated DEPs, such as E-selectin, gp130, Siglec-5 and PDGF-AA, are labeled on the left. **(B)** STRING analysis of the 40 DEPs (top 20 upregulated DEPs and top 20 downregulated DEPs). A total of three clusters were identified with known/predicted/other interactions. Cluster 1, represented with 26 red balls, and was centered on CXCL9. Cluster 2, represented with 9 green balls, was centered in INS.

**Figure 4:**
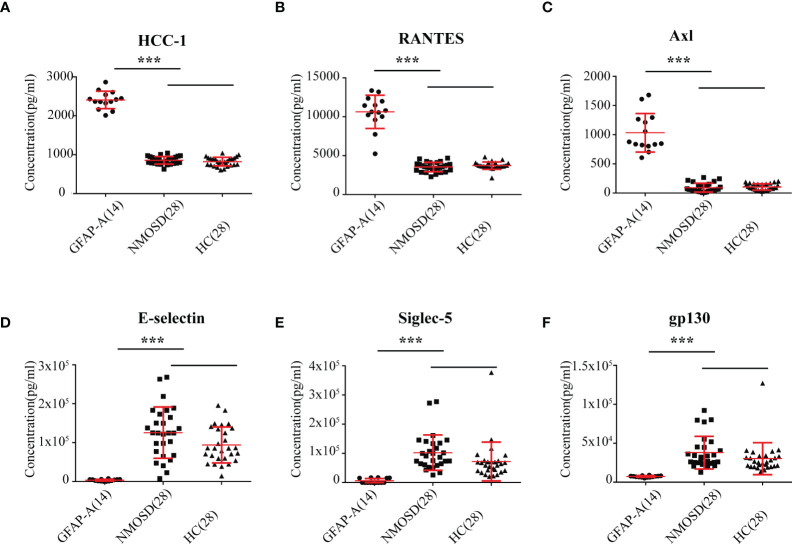
Scatter plot of the top 6 DEPs unique to GFAP-A. Serological concentrations (pg/ml) of the top 3 upregulated proteins (HCC-1, RANTES and Axl) that unique to GFAP were scatter plotted in **(A–C)**. Serological concentrations (pg/ml) of the top 3 downregulated proteins (E-selectin, Siglec-5 and gp130) that unique to GFAP-A patients were scatter plotted in **(D–F)**. The results are the means ± SEM. The non-parametric test was used for statistical analyses, and a p value of 0.05 or less was considered significant. ***p < 0.001.

Notably, we identified 127 DEPs between GFAP-A patients and HCs but not between NMOSD patients and HCs (**Figure 2A**, red box). There were 13 DEPs unique to NMOSD patients compared to HCs (**Figure 2A**, green box). The log10-transformed adjusted *p*-values of the top 40 DEPs unique to GFAP-A patients were scattered again. As shown in **Figure 3A**, IL-28A, VEGF R3, HCC-1, RANTES, and Axl were upregulated in GFAP-A serum, but TGF beta 2, IL-2Rg, Cripot-1, E-selectin, gp130, Siglec-5, and PDGF-AA were downregulated in GFAP-A serum. STRING analysis of cytokine profiles revealed that 40 DEPs unique to GFAP-A patients were interconnected (**Figure 3B**). Three clusters were identified, with 26 cytokines grouped together, but the other 9 cytokines and 4 cytokines formed another two separate clusters. The 26-cytokine cluster was primarily enriched in the activation-induced cell death of T cells, and the 9-cytokine cluster was primarily enriched in the regulation of the insulin-like growth factor receptor signaling pathway. The 4-cytokine cluster was enriched in viral protein interactions with cytokines and cytokine receptors (Additional BP and KEGG information is shown in **Supplementary Table 3**). The protein-protein interactions of 13 DEPs unique to NMOSD were showed in **Supplementary Figure 1B**. The BP and KEGG analysis results were showed in **Supplementary Table 4**. Scatter plots indicated the serological concentrations (pg/ml) of the top 3 upregulated cytokines (HCC-1, RANTES and Axl) and the top 3 downregulated cytokines (E-selectin, Siglec-5 and gp130) unique to GFAP-A patients in the respective study groups (**Figure 4**).

### Relationships between serum cytokines and anti-GFAP antibody titers

**Figure 5:**
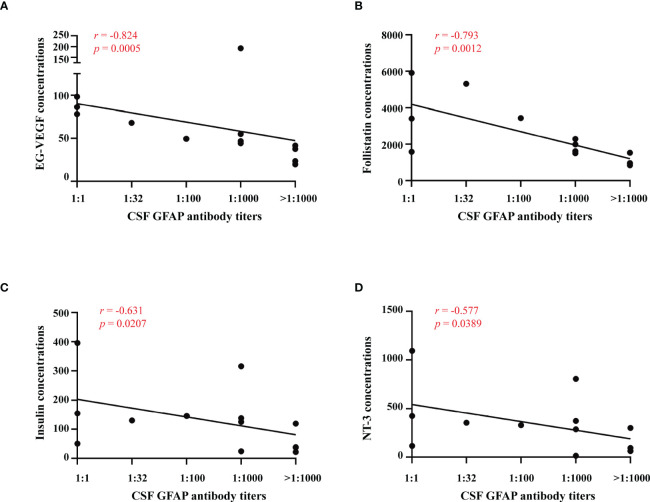
Relationships between serum cytokines and anti-GFAP antibody titers. Correlations between anti-GFAP antibody titer and serum levels of EG-VEGF, follistatin, insulin and NT-3 were analyzed by spearman’s rank analysis and were showed in **(A–D)**.

We diluted the CSF 1:10, 1:32, 1:100, 1:1000 and over 1:1000 to detect anti-GFAP antibody titers and analyzed the relationships between serum cytokines and anti-GFAP antibody titers in GFAP-A patients. The results showed that the anti-GFAP antibody titers significantly negatively correlated with EG-VEGF (*r* = -0.824, *p* = 0.0005), follistatin (*r* = -0.793, *p* = 0.0012), insulin (*r* = -0.631, *p* = 0.0207) and NT-3 (*r* = -0.577, *p* = 0.0389) (**Figure 5**).

### Relationships between serum cytokines and clinical severity

**Figure 6:**
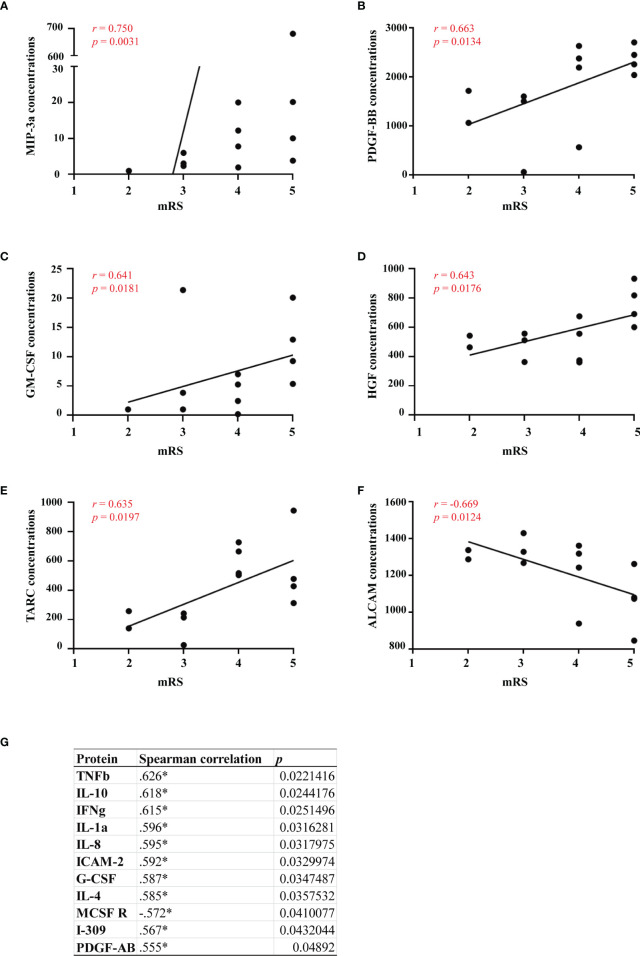
Relationships between serum cytokines and clinical severity. Correlations between clinical severity and serum levels of MIP-3a, PDGF-BB, GM-CSF, HGF, TARC, ALCAM and other proteins were analyzed by spearman’s rank analysis and were showed in **(A–G)**. *p<0.05.

To evaluate the relationships between serum cytokines and clinical severity in GFAP-A patients, we examined the correlations between them. The mRS scores significantly positively correlated with serological concentrations of MIP-3a (*r* = 0.750, *p* = 0.0031), PDGF-BB (*r* = 0.663, *p* = 0.0134), HGF (*r* = 0.643, *p* = 0.0176), GM-CSF (r = 0.641, *p* = 0.0181) and TARC (*r* = 0.635, *p* = 0.0197). The mRS scores significantly negatively correlated with ALCAM (*r* =-0.669, *p* = 0.0124) (**Figure 6**).

### Identification of correlations between CSF abnormalities and serum proteins in GFAP-A patients

**Figure 7:**
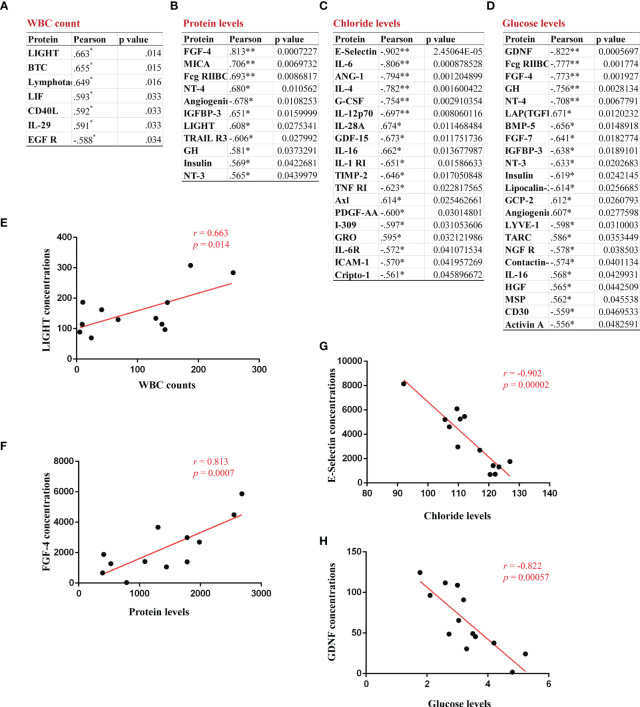
Identification of correlations between CSF abnormalities and serum proteins in GFAP-A patients. The serum proteins with a significant correlation with CSF levels of WBC count, protein, chloride and glucose are shown in **(A–D). (E)** A positive correlation between CSF WBC count and the serum concentrations of LIGHT. **(F)** A positive correlation between CSF protein levels and the serum concentrations of FGF-4. **(G)** A negative correlation between CSF chloride levels and the serum concentrations of E-selectin. **(H)** A negative correlation between CSF glucose levels and the serum concentrations of GDNF. Pearson’s correlation analysis was used for statistical analyses, *p<0.05, and **p<0.01 or less was considered significant.

To determine whether a signature of serum cytokines was associated with CSF abnormalities, we analyzed the relationship of 200 serum cytokine concentrations with the levels of WBCs, proteins, chloride and glucose in the CSF of GFAP-A patients. A significant positive correlation between CSF WBC levels and serum levels of LIGHT (*r* = 0.663, *p* = 0.014, **Figure 7E**), BTC (*r* = 0.655, *p* = 0.015), lymphotactin (*r* = 0.649, *p* = 0.016), LIF (*r* = 0.593, *p* = 0.033), CD40L (*r* = 0.592, *p* = 0.033) and IL-29 (*r* = 0.591, *p* = 0.033) was observed (**Figure 7A**). A significant positive correlation between CSF protein levels and serum levels of FGF-4 (*r* = 0.813, *p* = 0.0007, **Figure 7F**), MICA (*r* = 0.706, *p* = 0.0069), Fcg RIIBC (*r* = 0.693, *p* = 0.0086), NT-4 (*r* = 0.680, *p* = 0.0105), IGFBP-3 (*r* = 0.651, *p* = 0.0159), LIGHT (*r*=0.608, *p*=0.0275), GH (*r* = 0.581, *p* = 0.0373), insulin (*r* = 0.569, *p* = 0.0422) and NT-3 (*r* = 0.565, *p* = 0.0439) was observed. A significant negative correlation between CSF protein levels and serum levels of angiogenin (*r* = -0.678, *p* = 0.0108) and TRAIL R3 (*r* = -0.606, *p* = 0.0279) was observed (**Figure 7B**). Our results found that 16 serum cytokines were negatively correlated, and 3 cytokines were positively correlated between CSF chloride levels (**Figures 7C, G**, mostly correlated cytokine, E-selectin, *r* = -0.902, *p* = 0.00002). Our results showed that 16 serum cytokines were negatively correlated, and 7 cytokines were positively correlated between CSF glucose levels (**Figures 7D, H**, mostly correlated cytokine, GDNF, *r* = -0.822, *p* = 0.0005).

## Discussion

The present study identified a series of serological proteins that were differentially expressed between GFAP-A patients and NMOSD patients compared to HCs and verified a profile of serological DEPs that was unique to GFAP-A patients. We described the correlations between serological protein levels and CSF anti-GFAP antibody titers and clinical severity. We found that CSF anti-GFAP antibody titers significantly negatively correlated with EG-VEGF, follistatin, insulin and NT-3. Clinical severity (mRS scores) significantly positively correlated with MIP-3a, PDGF-BB, HGF, GM-CSF and TARC. CSF GFAP IgG is essential for diagnosis, but it is not a suitable biomarker. The serological proteins characterized in this study may be useful biomarkers for GFAP-A.

CCL5 (C-C motif chemokine ligand, also known as RANTES) is a chemokine that favors chemotactic signaling to T lymphocytes, basophils, and eosinophils in the peripheral immune system (12). Previous studies indicated that serum levels of CCL5 were significantly increased in multiple sclerosis (MS) patients (13) and in experimental autoimmune encephalomyelitis (EAE) animals (14). Another studies reported that levels of CCL5 increased dramatically when human immunodeficiency virus 1 (HIV-1) infection occurs (15) and at the onset and during the progression of MS (16). A previous study reported a positive correlation between the levels of CCL5 and the onset of synaptic glutamatergic alteration in EAE mice (17). The present study first found that serum levels of CCL5 in GFAP-A patients were significantly higher than NMOSD patients and HCs. However, whether elevated levels of CCL5 correlate with the pathogenesis of GFAP-A and whether the expression of CCL5 in the CNS is changed by GFAP-A are not clear. It may be necessary to us the GFAP-A animal model to explore the function of CCL5 in GFAP-A and determine whether the CCL5/CCR5 axis is a potential target for the treatment of GFAP-A.

C-C motif chemokine ligand 20 (CCL20), also known as MIP-3a and LARC, is involved in the recruitment of proinflammatory IL17-producing helper T cells (Th17) and regulatory T cells (Tregs) to sites of inflammation to regulate the progression of immune-mediated diseases (18). Previous studies reported that activated astrocytes in MS patients and in EAE mice secreted CCL20, which signals the continued recruitment of immune cells into the CNS (19, 20). Active and passive immunization against CCL20 improved EAE and are potential therapies for MS (21). A previous study showed that GFAP-A patients had significantly higher levels of CSF CCL20 than other patients (MS, NMOSD, and psychosomatic disorders), except patients with varicella zoster virus meningitis (4). One case-series study demonstrated that many GFAP-A patients experienced lymphocytic pleocytosis that lasted for several months, which may be due to elevated levels of CCL20 secreted by activated astrocytes (22). In our study, we found that the serum levels of CCL20 in GFAP-A patients were significantly higher than HCs and significantly positively correlated with mRS scores. This result suggests that serum CCL20 levels are a biomarker to assess clinical severity and may be a potential target for the therapy of autoimmune GFAP astrocytopathy.

Tumor necrosis factor superfamily (TNFSF) molecules play important roles in the activation, proliferation, differentiation, and migration of immune cells into the CNS. Our study found that the 20 DEPs in the GFAP and NMOSD patient groups were primarily enriched in the tumor necrosis factor-mediated signaling pathway and regulation of T-cell activation and were centered in CD40LG. CD40L also positively correlated with CSF WBC counts. The CD40 ligand (CD154, also known as TNFSF5) is expressed on activated T cells and binds to its cognate receptor CD40 (TNFRSF5). Disruption of CD40L-CD40 interaction inhibited clinical manifestations and ameliorated EAE in mice and monkeys (23, 24). The results suggested that GFAP and AQP4 astrocytopathy shares some common pathology related to TNF signaling.

Notably, our results showed that serum LIGHT was a DEP in the GFAP and NMOSD patient groups compared to HCs and positively correlated with CSF WBC counts and CSF protein levels in GFAP patients. The TNF superfamily ligand LIGHT (CD258, also known as TNFSF14) is a type II transmembrane glycoprotein that is expressed on activated T cells, NK cells, monocytes, granulocytes, and immature dendritic cells (25). LIGHT is a co-stimulator of T cells and stimulates a Th1 profile of cytokines, such as IFN-γ (26). LIGHT increased T-cell proliferation *in vitro* and induced apoptosis in thymocytes (27). A previous study showed that LIGHT-deficient mice developed more severe MOG_35-55_-induced EAE due to intensive activation of microglia/macrophages and an increased frequency of apoptotic cells with the CNS parenchyma compared to wild-type animals (28). Regarding the pathology of GFAP-A, an animal model study (29) and clinical results indicated that T-cell-mediated immunity played an important role in GFAP astrocytopathy (3). All of the results suggested that LIGHT played an important role in the pathogenesis of GFAP astrocytopathy by controlling the activated microglia/macrophages during CNS inflammation.

Besides to the serological biomarkers identified in the present study, previous studied have reported certain proteins that were changed in the CSF of GFAP-A patients. Akio et al. found that the expression levels of four cytokines (tumor necrosis factor alpha [TNFα], Interleukin [IL]-27, IL-6, and CCL20) and three biological markers (GFAP, S100 calcium-binding protein B, and neurofilament light chain [NFL]) were elevated in the CSF of acute phase of GFAP-A (4). Ying et al. reported that, compared to controls, CSF levels of Nod-like receptor protein 3 (NLRP3) inflammasome and inflammatory cytokines (IL-1 beta, IL-6 and IL-17) were significantly elevated in GFAP-A patients, and were positively related to the disease severity (30).

## Data availability statement

The original contributions presented in the study are included in the article/**Supplementary Material**. Further inquiries can be directed to the corresponding authors.

## Ethics statement

The committees for ethical review of research involving human subjects at the Second Affiliated Hospital of Guangzhou Medical University (Guangzhou, China) approved this study (Protocol Number: 2019-hs-11).

## Author contributions

C-CF and LH: contributed to the conception of the study, performed the experiment, collected the data and wrote the manuscript; L-FX, H-LL, and L-HJ: helped with the data collection and data analyses; SL, J-JY, and CL helped to experiments preparation and clinical information collection; X-GY and Y-ML designed the study, revised the manuscript and oversaw the entire project. All authors contributed to the article and approved the submitted version.

## Funding

This study was supported by China Postdoctoral Research Foundation (2021M690789), National Natural Science Foundation of China (81771302), Guangzhou Science and Technology Program key projects (202002020021).

## Acknowledgments

We are grateful for all patients who participated in this study.

## Conflict of interest

The authors declare that the research was conducted in the absence of any commercial or financial relationships that could be construed as a potential conflict of interest.

## Publisher’s note

All claims expressed in this article are solely those of the authors and do not necessarily represent those of their affiliated organizations, or those of the publisher, the editors and the reviewers. Any product that may be evaluated in this article, or claim that may be made by its manufacturer, is not guaranteed or endorsed by the publisher.

## Abbreviations

GFAP, autoimmune glial fibrillary acidic protein astrocytopathy; NMOSD, neuromyelitis optica spectrum disorder; MS, multiple sclerosis; EAE, experimental autoimmune encephalomyelitis; TNFSF, tumor necrosis factor superfamily; NFL, neurofilament light chain; NLRP3, nod-like receptor protein 3; EG-VEGF, endocrine-gland-derived vascular endothelial growth factor; TBA, tissue-based assay; CBA, cell-based assay; CSF, cerebrospinal fluid; DEPs, differential expression proteins; PPI, protein-protein interaction; KEGG, Kyoto Encyclopedia of Genes and Genomes; mRS, modified Rankin Scale; MRI, magnetic resonance imaging; MIP-3a/CCL20, C-C motif chemokine ligand 20; HGF, hepatocyte growth factor; GM CSF, granulocyte-macrophage colony-stimulating factor; TARC/CCL17, thymus and activation regulated chemokine; ALCAM/CD166, activated leukocyte cell molecule; LIF, leukemia inhibitory factor; FGF-4, fibroblast growth factor-4; RANTES/CCL5, C-C motif chemokine ligand.
