# Supplementary figures and images for: Serological biomarkers in autoimmune GFAP astrocytopathy
Source: Front Immunol. 2022 Aug 2;13:957361. doi: 10.3389/fimmu.2022.957361 (PMC9378990; doi:10.3389/fimmu.2022.957361)

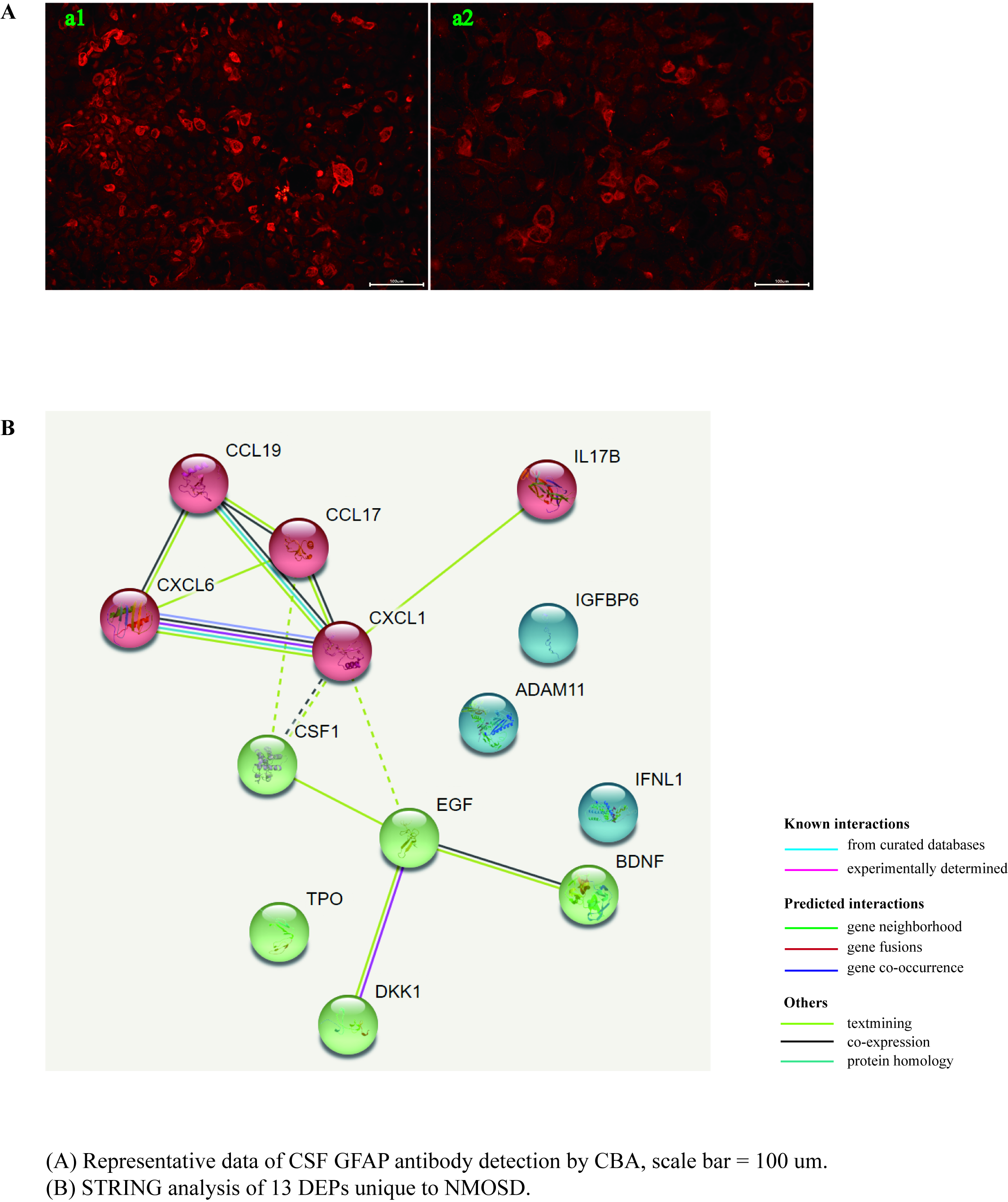

Supplement: Supplementary file 1 [file Image_1.tif]
